# The Use of Cashew Nut Shell Liquid (CNSL) in PP/HIPS Blends: Morphological, Thermal, Mechanical and Rheological Properties

**DOI:** 10.3390/ma12121904

**Published:** 2019-06-13

**Authors:** Mirna Nunes Araújo, Leila Lea Yuan Visconte, Daniel Weingart Barreto, Viviane Alves Escócio, Ana Lucia Nazareth da Silva, Ana Maria Furtado de Sousa, Elen Beatriz Acordi Vasques Pacheco

**Affiliations:** 1 Instituto de Macromoléculas Professora Eloisa Mano, Universidade Federal do Rio de Janeiro, 2.300 Horácio Macedo Av., Technology Center, Building J, 21941-598 Rio de Janeiro, RJ, Brazil; mirnana9@hotmail.com (M.N.A.); lyv@ima.ufrj.br (L.L.Y.V.); vivi75@ima.ufrj.br (V.A.E.); ananazareth@ima.ufrj.br (A.L.N.d.S.); 2 Programa de Engenharia Ambiental (PEA/UFRJ), Universidade Federal do Rio de Janeiro, 149 Athos da Silveira Av., Technology Center, Building J, 21941-909 Rio de Janeiro, RJ, Brazil; 3 Escola de Química, Universidade Federal do Rio de Janeiro, 149 Athos da Silveira Av., Technology Center, Building E, 21941-611 Rio de Janeiro, RJ, Brazil; dbarreto@eq.ufrj.br; 4 Department of Chemical Processes, Universidade do Estado do Rio de Janeiro, 524 São Francisco Xavier, 20550-900 Rio de Janeiro, RJ, Brazil; ana.furtado.sousa@gmail.com

**Keywords:** polymer mixtures, blends, cashew nut shell liquid (CNSL), polypropylene, high impact polystyrene, compatibilization

## Abstract

Polypropylene (PP) and high impact polystyrene (HIPS) are two polymers that are frequently found in disposable waste. Both of these polymers are restricted from being separated in several ways. An easier way to reuse them in new applications, without the need for separation, would require them to be less immiscible. In this work, cashew nut shell liquid (CNSL), a sub-product of the cashew agroindustry, was added as a third component to PP-HIPS mixtures and its effect as a compatibilizing agent was investigated. Morphological results showed that CNSL acted as an emulsifier by promoting reduction in the domains of the dispersive phase, HIPS, thus stabilizing the blends morphology. Differential scanning calorimetry (DSC) analysis suggests that CNSL is preferably incorporated in the HIPS phase. Its plasticizing effect leads to more flexible materials, but no significant effect could be detected on impact resistance or elongation at break.

## 1. Introduction

The practice of recycling through reprocessing can reduce the volume of waste in landfills, simultaneously generating a new economic activity, saving energy and non-renewable resources [1,2]. The mechanical recycling is based on the conversion of such residues, as post-industrial or post consumption plastic materials into pellets through extrusion reprocessing. This procedure allows the subsequent use of the pellets in the production of various products such as garbage bags, soles, floors, hoses, car components, non-food packaging, etc. [3].

A limitation to mechanical recycling is the heterogeneous composition of the residues, which are formed by different types of plastic materials that are usually incompatible. When mixed, the incompatible materials give rise to products of poor mechanical performance.

The wide consumption of disposable products, as dishes and cups, heavily contributes to the total volume of discarded urban plastic, since they are rapidly discarded after a very short period of usage. These artifacts are very similar regarding their appearance; unlike polyethylene terephthalate (PET) bottles, which are produced exclusively from PET, disposable dishes and cups can be manufactured either from polypropylene (PP) or from high impact polystyrene (HIPS) [4]. Thus, as these two polymers are incompatible, their mixtures give rise to phase separation and, as a consequence, to materials with poor properties [5]. Thus to be recycled, the previous separation of each post-consumer polymer should be carried out. So, despite the frequency with which they appear in urban garbage, these products are not potentially interesting from technical and economic points of view.

On the other hand, a proposal of processing blends of HIPS and PP without having to proceed to the separation step would allow a more favorable implementation of recycling for these artifacts. However, to succeed in obtaining PP/HIPS mixtures with good properties, a third component must be added in order to overcome the incompatibility between the two polymers. Thus, the use of an appropriate and economically viable compatibilizing agent should be considered. This has been the goal of a number of investigations.

Santana and Manrich [6] evaluated the efficiency of SEBS (styrene-ethylene-co-butylene-styrene copolymer) as a compatibilizing agent in PP/HIPS mixtures of different contents. They found that the incorporation of SEBS leads to more homogeneous morphologies, since the HIPS particles size was reduced and better dispersed throughout the PP matrix. The efficiency of an analogous polymer, SBS (styrene-butadiene-styrene tri-block copolymer) was also evaluated as compatibilizing agent for PP/HIPS by Fernandes et al. [7]. The influence of HIPS on the photodegradation of reprocessed PP was evaluated. It was observed that on increasing HIPS concentration from 10 to 30% *w*/*w*, the degradation effect on PP decreased. Another report by Fernandes et al. [8] addressed the influence of reprocessing on the degree of degradation by UV radiation. They observed that the resistance to radiation was lowest for virgin PP and its blends, and better resistance was related to those mixtures based on reprocessed PP.

The behavior of HIPS mixtures with low amounts of PP has also been studied. Parres et al. [9] determined thermomechanical and morphological properties of HIPS/PP blends in which the amount of PP varied from 2.5 to 10% *w*/*w*, in order to simulate the effect of occasional impurities on the properties. The authors observed that on increasing the content of PP in the mixture, mechanical properties were generally lower due to the increasing incompatibility between the polymers.

In this work, the use of cashew nut shell liquid (CNSL), a sub-product from the cashew nut industry, was evaluated as a compatibilizing agent in PP/HIPS blends [10]. As most commercial compatibilizing agents come from fossil resources, the industrial use of CNSL would represent an economic as well as a sustainable alternative.

In the cashew nut industry, the main product is the nut. CNSL is extracted from the husk by solvent extraction at high (180 to 200 °C) or low temperatures. At low temperatures the extraction product is called natural CNSL. At high temperatures, anacardic acid undergoes decarboxilation, giving rise to which is called technical CNSL. Thus, natural and technical CNSL have different compositions, as seen in Table 1 [11].

**Figure 1:**
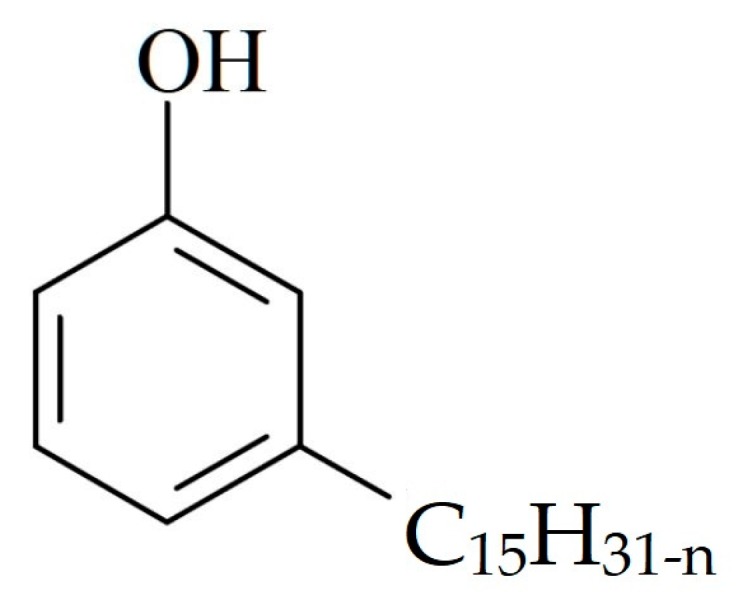
Chemical structure of cardanol; n depends on the number of unsaturations [12].

Technical grade CNSL is a mixture of phenols that combine the aromatic character to a long aliphatic, unsaturated chain. The major constituent is cardanol, which can be present in amounts varying from 68% up to 95% [12]. Its chemical structure is shown in Figure 1. CNSL constituents can be separated and purified, but the percentages obtained are relatively low and the cost of reagents and solvents is relatively high. In addition, contaminations with cardol and polymeric materials may prevent the large-scale production of cardanol [11].

CNSL is a biologically based lipid that is low cost, abundant and comes from a renewable resource. Its peculiar structure gives CNSL the possibility of a wide range of applications, including acting as a substitute for petroleum-based compounds [11,13,14]. The phenolic character allows CNSL to be a non-toxic anti-oxidant and raw material for phenolic and epoxy resins and plasticizers. It is also used as the basis for a number of industrial applications including adhesives, coatings and resins [15,16].

## 2. Materials and Methods

### 2.1. Materials

In this work, technical CNSL was provided by Irmãos Fontenele S.A.—Comércio, Indústria e Agricultura, Fortaleza, Ceará, Brazil. Isotactic polypropylene H605 (thermoforming grade), Melting Flow Index (MFI) = 2.1 g/10 min (230 °C, 2.16 kg) and density = 0.905 g/cm^3^, was provided by Braskem (Triunfo, Brazil) S.A. High impact polystyrene R 870E (6.2% polybutadiene), MFI = 4g/10 min (200 °C, 5 kg) was provided by Innova S.A. The solvents for the selective extraction were chloroform P.A. from Merck (Rio de Janeiro, Brazil) S.A and acetone P.A. from Sigma-Aldrich Brazil Ltd. (São Paulo, Brazil).

### 2.2. Preparation and Injection Molding of the Blends

An interpenetrating co-rotating twin screw extruder, model DCT-20 Teck Tril, screw diameter 20 mm and L/D = 36 was used. The temperature profile along the cylinder is seen in Table 2. The screw profile consisted in five KB45 kneading elements at compression zone, assuring the complete fusion of the polymer and leading to a good mixture between the blend components. The composition of mixtures comprised of PP, HIPS and CNSL was varied according to Table 3. The filaments coming out of the extruder passed through a Brabender pelletizer, under the conditions set by the equipment manufacturer.

The blends were injection molded in an Arburg Allrounder, model 270S-400-170, with 30 mm diameter and L/D = 20, into specimens for the analyses. Temperature profile and pressure, shown in Table 4, were selected as to preserve as much as possible the CNSL integrity. The operation procedure followed the ASTM D3641 (2012) standard.

### 2.3. Scanning Electron Microscopy (SEM)

For this test, the samples were immersed in liquid nitrogen and then fractured. As neither PP nor HIPS have any special chemical feature in the chains but just carbon and hydrogen, it was impossible to apply X-ray Dispersive Energy Spectroscopy (EDS) to dye one of the phases. Due to this, the samples related to the blends were immersed in chloroform for 4 h at 23 °C to promote the extraction of the HIPS phase and CNSL and allow a better visualization of phase distribution in the polymer blends. The samples surfaces were then metalized with gold and taken to the microscope (JEOL, model 1065-LV, Tokyo, Japan). Images were obtained from a secondary electron detector with a voltage acceleration of 20 kV.

### 2.4. Thermal Analysis by Differential Scanning Calorimetry (DSC)

DSC analyses were carried out on the unmixed resins, CNSL and the different mixes, under nitrogen. The equipment was a NETZSCH, model 204 F1 Phoenix (Selb, Germany). Sample weights in the range 7.5 to 8.5 mg were used, as suggested by ASTM E793. The first heating cycle was done at 20 °C/min, from room to 200 °C, kept at that temperature for 2 min, followed by cooling to 0 °C to rest for 5 min. The second heating was performed from 0 °C to 200 °C at a rate of 10 °C/min. The values used in the study were obtained after the second heating.

### 2.5. Mechanical Tests

The Izod impact resistance was measured following ASTM D256 (2010), in a CEAST Resil Impactor Tester (Pianezza, Italy) at room temperature. To proceed to the test, the sample was fixed vertically and submitted to a 2J hammer released from an angle of 150°.

The flexural modulus was determined by EMIC DL-3000 Universal Machine (São José dos Campos, Brazil), according to ASTM D790 (2015) with loading cell of 1000 kgf. The appropriate rate for the test was set at 1.4 mm/min, according to the aforementioned standard ASTM.

### 2.6. Rheological Measurements

The dynamic melt rheological measurements were performed using an oscillatory rheometer (AR 2000. TA Instruments with a parallel-plate geometry D = 25 mm). All tests were conducted at 200 °C. The linear viscoelastic zone was assessed by performing strain sweep tests from 0.1% to 100% at 1 Hz. Frequency sweep tests from 0.01 to 600 rad/s were subsequently performed to determine the dynamic properties of the materials at 5% strain under nitrogen atmosphere. The rheological behaviors of the samples were evaluated based on their complex viscosity (η*) and storage modulus (G’) as a function of frequency (ω).

## 3. Results

### 3.1. Morphology

**Figure 2:**
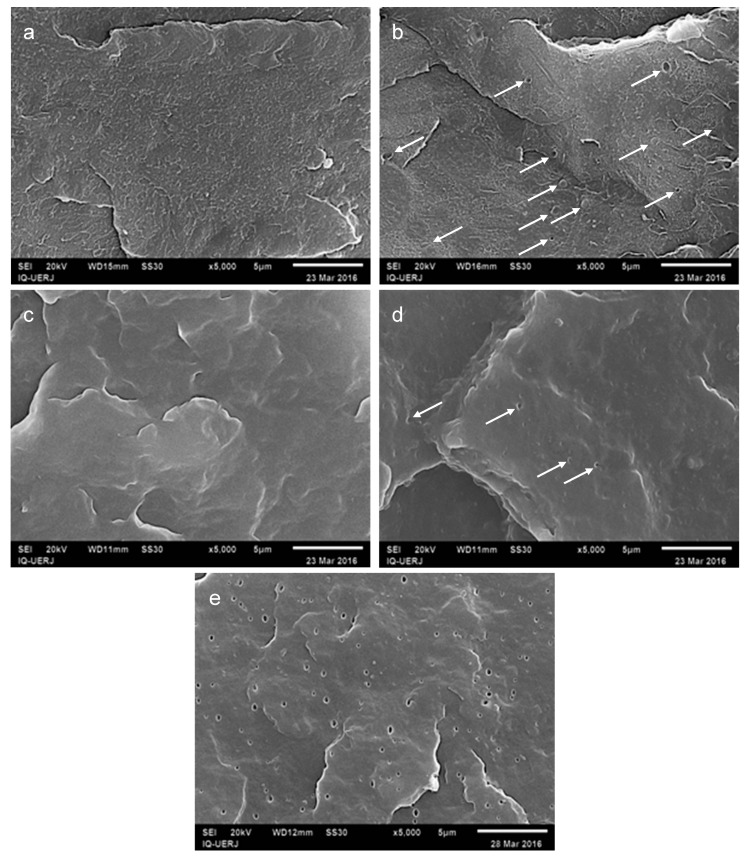
SEM micrographs of neat resins: (**a**) HIPS (without CNSL); (**b**) HIPS with 5 phr CNSL; (**c**) PP (without CNSL); (**d**) PP with 5 phr CNSL; (e) PP with 5 phr of CNSL, after chloroform extraction; 5000× amplification (5 μm scale).

Figure 2a–e shows SEM micrographs for neat PP and HIPS and the mixtures of each of these polymers with 5 phr of CNSL. Figure 2e also shows the mixture PP-CNSL 5 phr after CNSL extraction with chloroform. In Figure 2a,c for the neat polymers, PP seems to have a more homogeneous and smoother surface than HIPS, which presents a rougher surface. Such an aspect results from the typical “salami” type morphology of HIPS, since in the production of this material PS is obtained in the presence of polybutadiene (PB). The presence of PB during styrene polymerization leads to grafting and intercrossing of this rubber in such a way that PS domains are involved inside the tenacifier phase, thus giving rise to the “salami” morphology [17].

**Figure 3:**
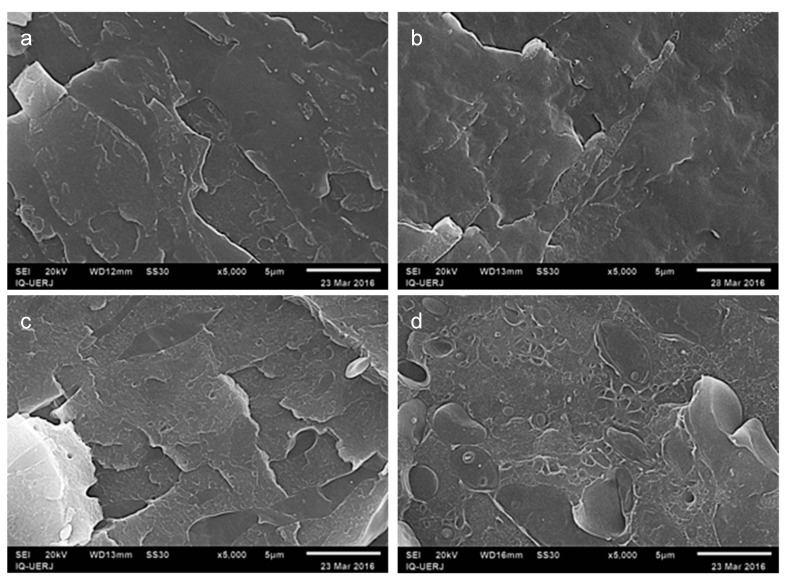
SEM micrographs for the blends PP/ HIPS: (**a**) 4:1 without CNSL; (**b**) 4:1 with 5 phr of CNSL; (**c**) 1:4 without CNSL; (**d**) 1:4 with 5 phr of CNSL. Magnification 5000×.

In Figure 2b and Figure 3d the effect of the addition of CNSL to either HIPS or PP, respectively, can be seen. Cavities and spherical particles appeared in HIPS micrograph, Figure 2b, which can be related to a localized concentration of CNSL. Figure 2d, on the other hand presented a more subtle difference. However, it can be observed that the typically smooth surface started to present slight roughness after the introduction of CNSL. To corroborate the hypotheses the sample was submitted to chloroform extraction for 4h, for the extraction of CNLS.

The result of this extraction is shown in Figure 2e, from which the holes formed due to the chloroform extraction of CNSL can be clearly observed. In the case of HIPS, as this polymer is soluble in chloroform and also in other common solvents for CNSL, the same strategy could not be employed.

In Figure 3 the micrographs refer to PP/HIPS 4:1 without CNSL (Figure 3a) and with 5 phr of CNSL (Figure 3b). Rough regions related to HIPS phase dispersed in the homogeneous PP matrix can be observed. In addition, on comparing the two images, a reduction of HIPS domains brought about by the addition of CNSL to the mixture is suggested. Nevertheless, the presence of CNSL is not explicitly evidenced, except for the slightly rougher appearance of PP phase.

Likewise, micrographs were taken for 1:4 blends without CNSL (Figure 3c) and with 5 phr CNSL (Figure 3d), for comparison. In both cases, the images point to HIPS playing the role of the matrix and PP being the disperse phase. However, there is an explicit dissimilarity between the two morphologies due to the apparent increase in the roughness of the HIPS phase due to the emergence of more prominent notches and recesses not observed in the image related to the blend without CNSL.

In addition, the shape of PP domains has also undergone visible modifications, since the dispersed phase started to show a more circular appearance after CNSL insertion, indicating a more stable morphology than the original one.

This result suggests the possibility of forming a more favorable scenario, in which the oil would position itself at the interface of the two phases, thus reducing the tension between them and improving the properties of the blend. After that, all the 12 blends were immersed in chloroform for extraction of the HIPS phase, as well as the CNSL.

**Figure 4:**
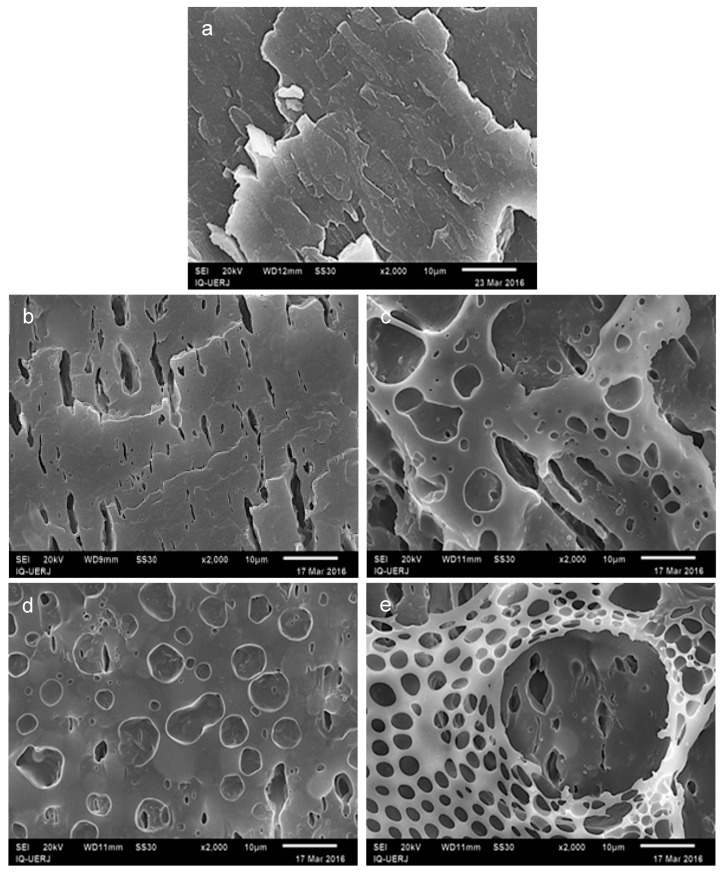
SEM micrographs for PP/HIPS 4:1 without CNSL, before extraction (**a**), and after extraction (**b**–**e**): without CNSL (**b**), with 2.5 phr CNSL (**c**), and with 5 phr CNSL at different regions of the sample (**d**,**e**). Amplification 2000× (10 μm scale).

Figure 4 shows micrographs for PP/HIPS 4:1 without CNSL, before extraction (Figure 4a), and after extraction (Figure 4b), the samples with 2.5 phr CNSL, after extraction (Figure 4c), and two different regions of sample with 5 phr CNSL, after extraction (Figure 4d,e).

Figure 4a shows that even with an amplification of 2000×, it is not possible to identify the two phases for the blend 4:1/0. When the sample is submitted to chloroform extraction, regions previously occupied by HIPS became observable, Figure 4b, characterized by cavity formation, deep and narrow, throughout the surface. It can be seen from these micrographs that the domains of the dispersed phase present a variety of dimensions, emphasizing the instability of the system. As for the stretched shape of these domains, one can presume that they have arisen during the injection molding process. In the process the melt material is injected under pressure into a cooled mold, which can provide the material with a processing dependent morphology.

Figure 4c–e corresponds to the 4:1 blends with 2.5 and 5 phr of CNSL. The images show morphological aspects which can be attributed to the presence of CNSL, since they do not appear in sample without CNSL. Specifically, in Figure 4d (4:1 blend with 5 phr CNSL, after extraction) aside the cavities coming from the extraction of HIPS, the appearance of concave and rounded depressions was also clearly observed, with the formation of circles in PP matrix. This morphology, nevertheless, cannot be attributed exclusively to the presence of CNSL since this pattern was not seen in the micrograph of neat PP+CNSL, after extraction. Therefore, cavity formation would be a combined effect of both CNSL and HIPS being present in the PP matrix.

However, the same blend 4:1/5, under the analysis of a different spot (Figure 4e), revealed a peculiar morphology type that is different from that showed in Figure 4d. In Figure 4e, a perforated film can be seen on the material surface that has not been observed before. The same pattern appears on a smaller scale in Figure 4c (4:1/2.5), where round and stretched cavities are seen. Such an unusual morphology motivated a deeper investigation that will be described later on.

**Figure 5:**
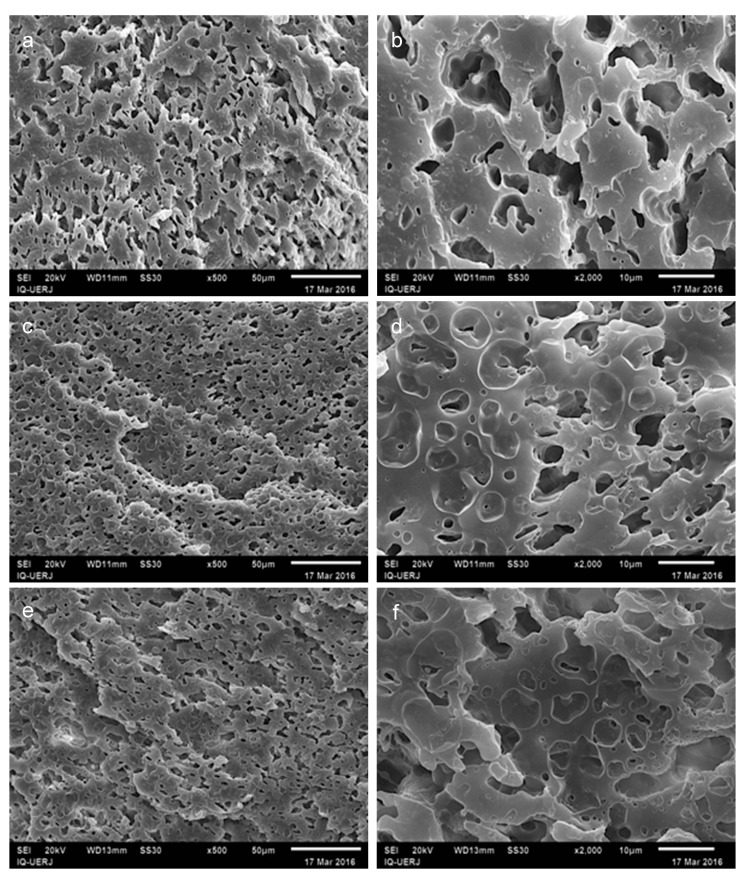
SEM micrographs of 3:2 PP/HIPS blends, after chloroform extraction: (**a**,**b**) without CNSL; (**c**,**d**) with 2.5 phr of CNSL; (**e**,**f**) with 5.0 phr of CNSL; images on left side, magnification of 500×; images on right side magnification of 2000×.

Blends 3:2 were also evaluated after chloroform extraction of HIPS phase and their SEM images are presented in Figure 5 at two magnifications. On the left side the images were taken at 500× and on the right side, at 2000× magnification. Figure 5a,b shows micrographs of 3:2/0 blend where the evident increase of the HIPS domains are seen, when compared with 4:1/0 blend (Figure 5b) due to the doubled amount of this polymer in the mixture.

Figure 5c,d, related to 3:2 PP/HIPS blends with 2.5 phr of CNSL, and Figure 5e,f corresponding to the blends with 5phr of CNSL, present a clear reduction in domain size associated to the previous localization of HIPS, in addition to a higher homogeneity of these sizes, suggesting a more stable morphology, as compared with the blend with no CNSL.

Moreover, it was also possible to verify the occurrence of circles in the PP matrix, as seen before in the micrograph of 4:1 blend containing CNSL (Figure 4d), thus corroborating the hypothesis that the formation of these cavities is intrinsically associated to the insertion of CNSL in PP/HIPS mixtures.

**Figure 6:**
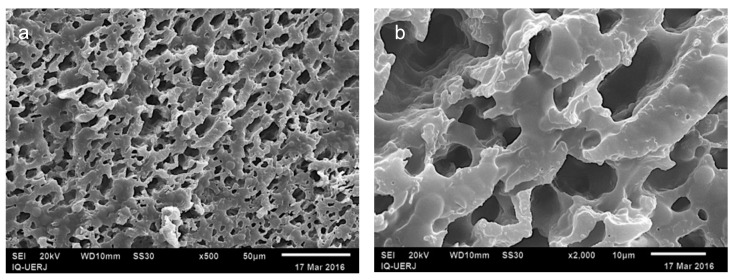

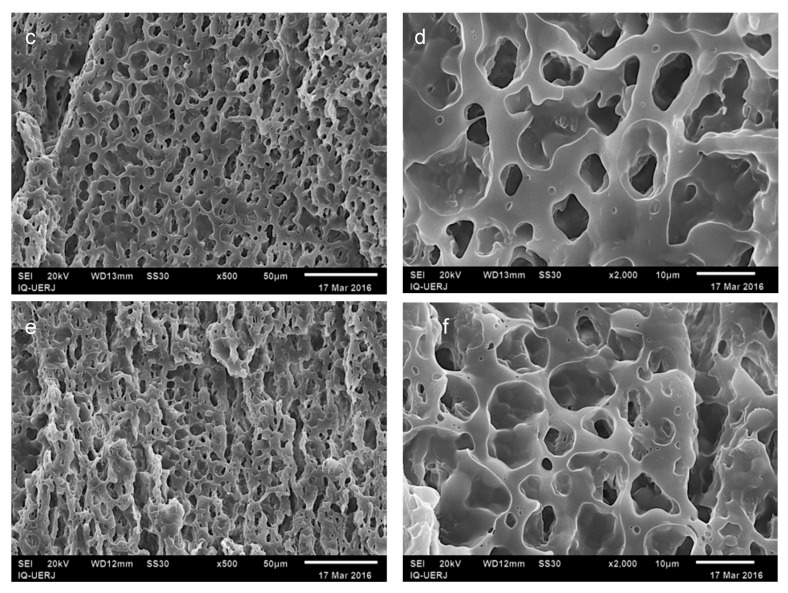
SEM micrographs of 2:3 blends, after chloroform extraction: (**a**,**b**) without CNSL; (**c**,**d**) with 2.5 phr of CNSL; (**e**,**f**) with 5.0 phr of CNSL; images on left side with 500× magnification; images on right side with 2000× magnification.

Going further with SEM analysis, Figure 6 presents the micrographs for 2:3 blends. In Figure 6a,b, as expected, the number of cavities related to HIPS domains tends to increase as the amount of this polymer in the blends also increases. Nevertheless, in spite of HIPS accounting for 60% of the total mass, it is still possible to verify continuity in the remaining phase (PP). This tendency of PP to play the role of the matrix when in blends with polystyrene (PS) has been widely studied by Omonov et al. [18]. The authors used selective dissolution experiments to quantitatively estimate the total continuity of PS phase in PP/PS compositions. They found that it occurs between 70%/30% and 10%/90% (*w*/*w*), depending on the viscosity ratio of the constituents.

It is worth noting that the image seen in Figure 6a,b suggests that morphology is very close to a co-continuous one. However, on the addition of CNSL to the mixtures, a reduction of HIPS domains took place (Figure 6c–f), inhibiting the occurrence of a co-continuous system in these compositions. On the other hand, the reduction in the disperse domains as well as the better homogeneity in the morphology provide better properties to the blends due to the stabilizing effect of CNSL.

**Figure 7:**
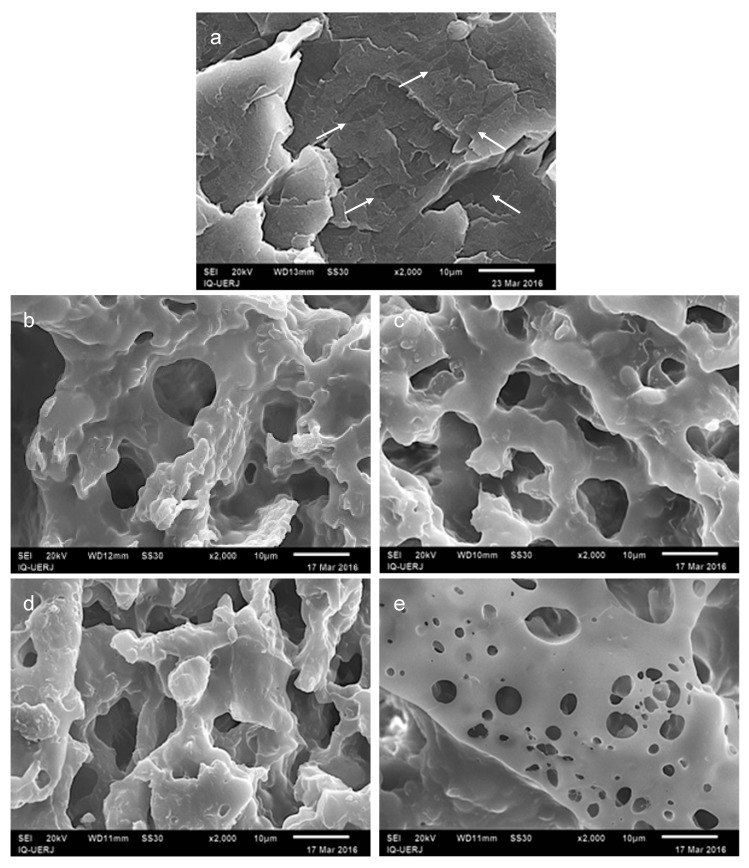
SEM micrographs of 1:4 PP/HIPS blends; before chloroform extraction: (**a**) without CNSL; after chloroform extraction: (**b**) no CNSL, (**c**) with 2.5 phr of CNSL, (**d**) and (**e**) different aspects of blend with 5 phr of CNSL. Magnification of 2000×.

Figure 7 shows the micrographs for the 1:4 PP/HIPS mixtures, before and after chloroform extraction, at 2000× magnification. In Figure 7a, PP domains dispersed in the HIPS matrix can be identified, suggesting that in the 1:4 blends without CNSL, a co-continuous morphology has been achieved. If this is so, it can be said that for a PP/HIPS system, in the absence of CNSL and under the imposed processing conditions, a co-continuous morphology can be formed when components ratios vary between 2:3 and 1:4, that is, between 40%/60% and 20%/80% *w*/*w*.

After selective extraction of HIPS (Figure 7b), the 1:4/0 blend presented cavities and deep cracks, in addition to entire regions which have been extracted, an indicative of the effective occurrence continuity of the HIPS phase. However, the remaining PP, also observed in the images, shows the concomitant occurrence of continuity of this phase, most probably partial.

In Figure 7c,d more homogeneous morphologies than that for the blend without CNSL (Figure 7b) are observed. Also, the images presented a visual aspect typical of co-continuous systems, which may suggest a morphological stability much superior to that for the 1:4/0 blend.

**Figure 8:**
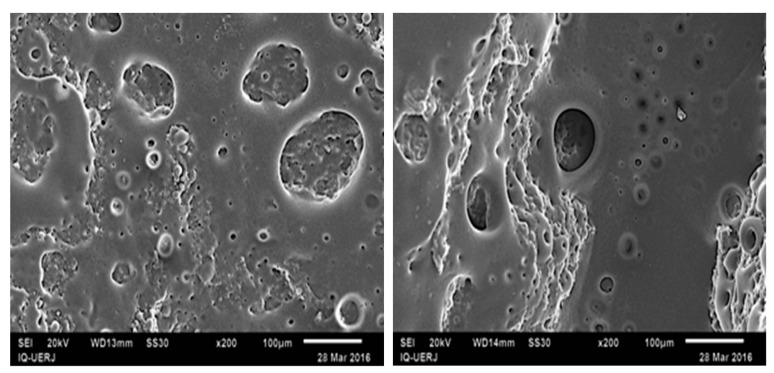
SEM Micrographs of 4:1 PP/HIPS blend with 5 phr of CNSL after acetone extraction of HIPS phase in two different regions of the same sample. Magnification 200×.

Figure 7e shows another region of the 1:4/5 blend where a perforated film is again observed, as in Figure 4e. With the purpose of clarifying the casual formation of this atypical morphology, another selective extraction of the HIPS phase was carried out, this time with acetone instead of chloroform, during the same period of time. Since acetone is not a solvent as good as chloroform for HIPS, the extraction was only partial, leaving HIPS residues on the surface of the sample as seen in Figure 8.

On comparing with the atypical morphologies observed after the extraction with chloroform, a similarity in the pattern is found, thus suggesting that the perforated formation would be caused by some remaining amounts of HIPS on the sample surface due to an incomplete HIPS dissolution by chloroform. As this morphology was observed only in those blends with CNSL, one can infer that the oil may have somehow interfered with the HIPS solubility in chloroform.

It is worth noting, as previously seen in Figure 4d which shows the 4:1 blend with 5 phr of CNSL, that a different morphology of HIPS phase was found which suggests that the presence of CNSL in the mixture could be the key for the formation of the peculiar aspect of a perforated film.

### 3.2. Thermal Behavior

From DSC curves polymer transition temperatures as well as heat flow and degree of crystallinity were determined. Glass transition temperatures were taken from the inflection point and a deviation of 2 °C was considered, as suggested by ASTM E1356 (2014). The same procedure was followed for the determination of melting enthalpy according to ASTM E793 (2012).

The degree of crystallinity was determined from the endothermic peak relative to the enthalpy of crystalline melting, calculated by Equation (1) [19].

(1) $$X_{c}\left(\%\right)=\frac{∆H_{f}}{∆H_{f}^{o}}\times 100$$

where, $X_{c}$ is the degree of crystallinity of the sample; $∆H_{f}$ is the variation in the melting enthalpy of the sample, measured by the area of the melting peak in the curve obtained by DSC; $∆H_{f}^{o}$ is the variation in the enthalpy of melting for a 100% crystalline sample (determined by extrapolation).

As polystyrene is amorphous, the final crystallinity of the PP/HIPS blends can be attributed exclusively by the semi-crystalline polypropylene. The theoretical value of melting enthalpy for a 100% crystalline PP, used in the calculations was 207 J/g [20].

Concerning the samples with CNSL, a correction had to be made to compensate for the presence of the added oil. Thus, as suggested by ASTM E793, a standard deviation of 7.8% was used to correct the values of melting enthalpy, from now on referred to as $∆H_{f}^{c}$. To perform the analysis the protocol described in the experimental part was used.

**Figure 9:**
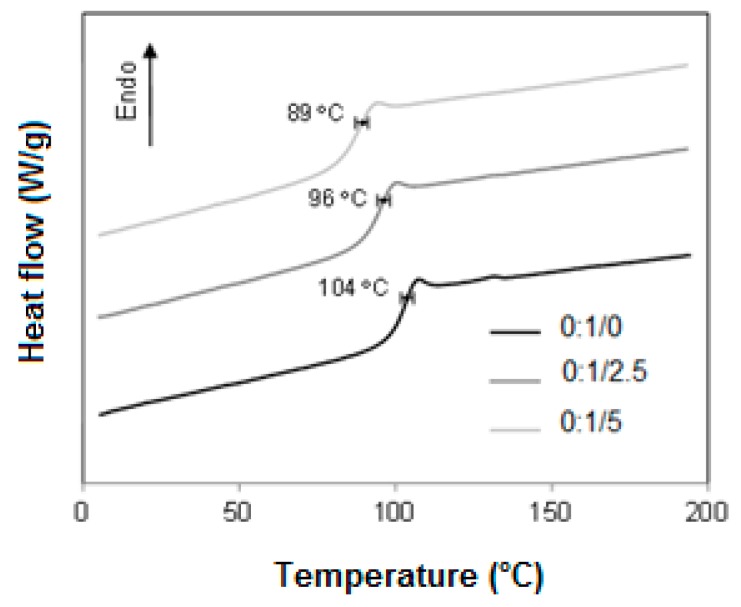
DSC thermograms for HIPS with 0, 2.5 and 5 phr of CNSL.

Figure 9 shows the thermograms related to HIPS with 0, 2.5 and 5.0 phr of CNSL. Looking at the curves it can be seen that a reduction in the glass transition temperatures occurs from 104 ± 2 °C to 96 °C, and then to 89 °C as the addition of CNSL increases from 0 to 5 phr. This can be credited to the role of CNSL as a plasticizer for the HIPS matrix, which contributes to chain mobility, allowing the amorphous phase of the polymer to flow at lower temperatures.

**Figure 10:**
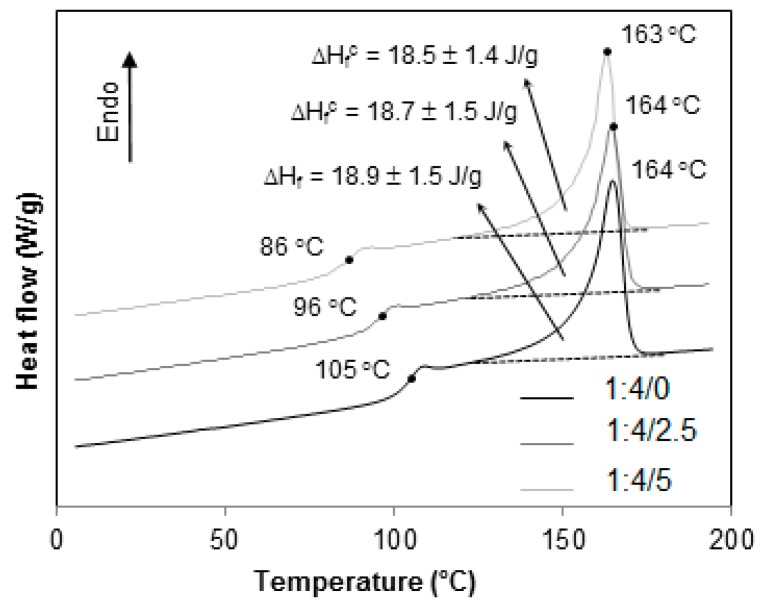
DSC thermograms for 1:4 PP/HIPS blends with 0, 2.5 and 5 phr of CNSL.

The thermograms in Figure 10 correspond to the blends PP/HIPS 1:4, with 0, 2.5 and 5 phr of CNSL. Again, the reduction in Tg is observed. However, the crystalline enthalpy of melting did not show any change.

**Figure 11:**
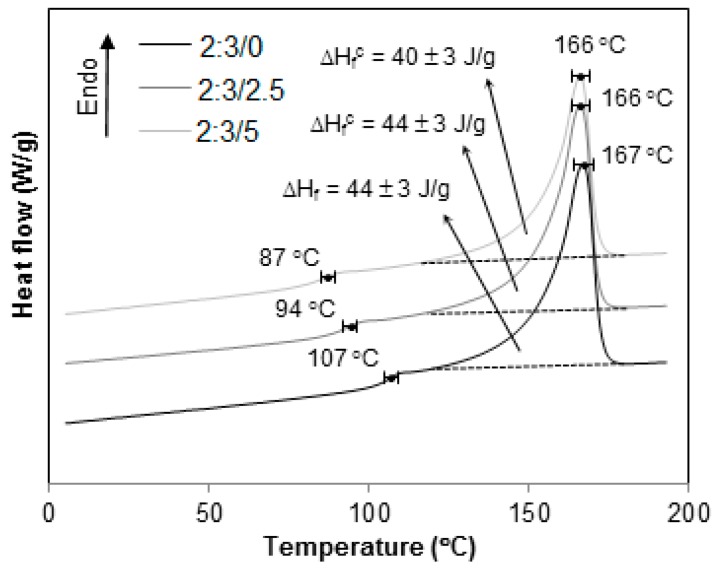
DSC thermograms for 2:3 PP/HIPS blends with 0, 2.5 and 5 phr of CNSL.

**Figure 12:**
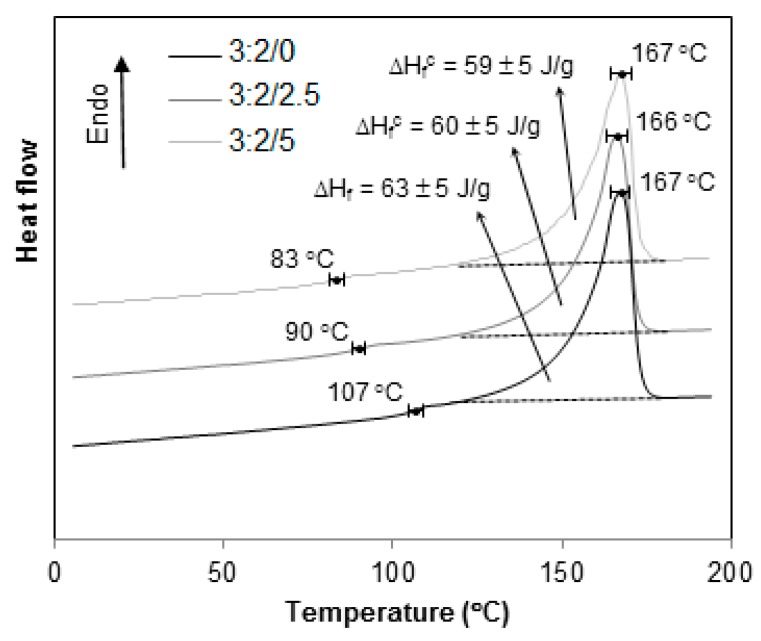
DSC thermograms for 3:2 PP/HIPS blends with 0, 2.5 and 5 phr of CNSL.

**Figure 13:**
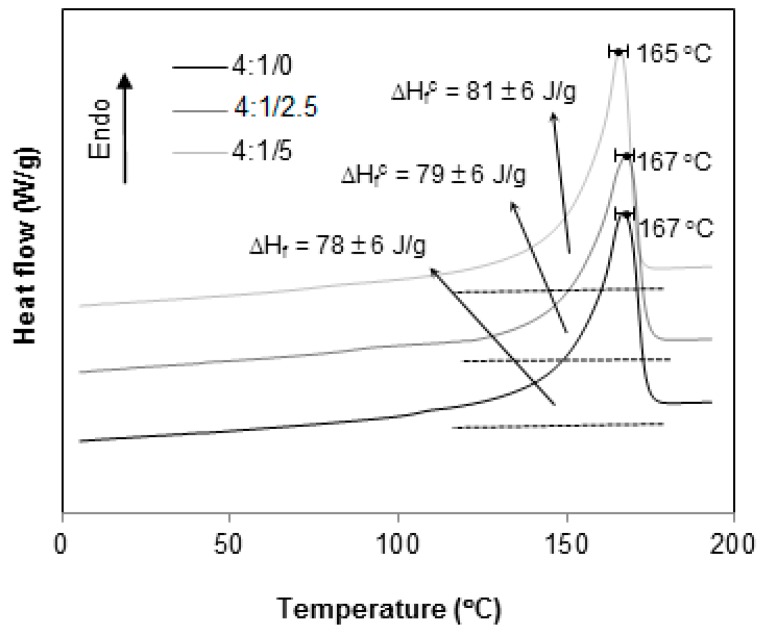
DSC thermograms for 4:1 PP/HIPS blends with 0, 2.5 and 5 phr of CNSL.

However, as the HIPs content decreases, Figure 11, Figure 12 and Figure 13, the signal associated to T_g_^HIPS^ becomes less and less visible till its total disappearance, as seen in Figure 13 for 4:1 blends.

**Figure 14:**
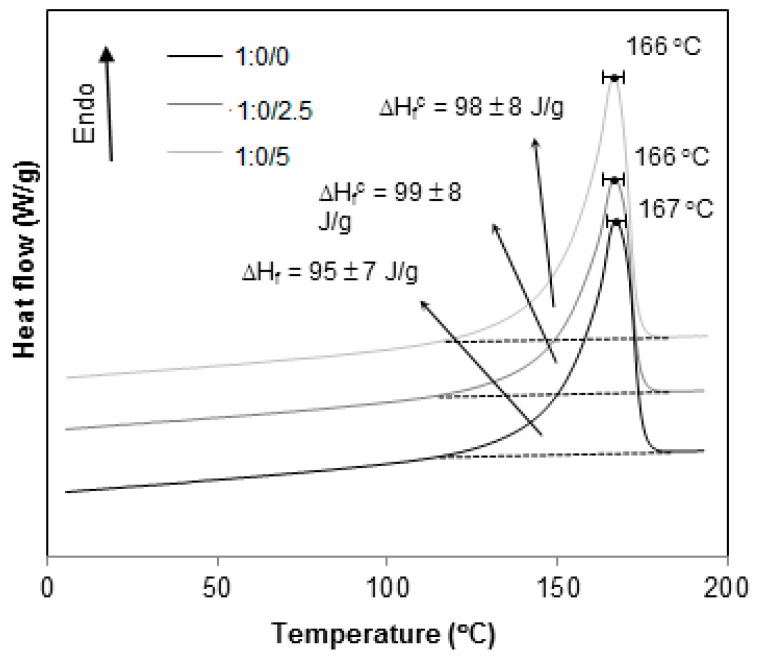
DSC thermograms of PP with 0, 2.5 and 5 phr of CNSL.

**Figure 15:**
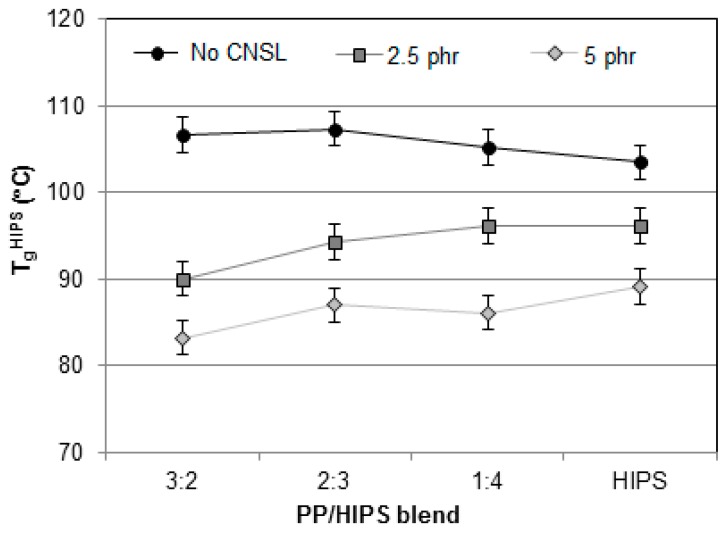
Tg values for HIPS and the 3:2, 2:3 and 1:4 PP/HIPS blends with 0, 2.5 and 5 phr of CNSL.

The thermograms in Figure 10, Figure 11, Figure 12, Figure 13 and Figure 14 present the curves for all blends, 1:4, 2:3, 3:2, 4:1 and 1:0, respectively. In general, the same tendency of T_g_^HIPS^ decrease as the CNSL content increases is observed. This behavior can be better visualized in Figure 15. In this figure, data for 4:1 blends were dismissed for lack of resolution in T_g_^HIPS^ determination. The decay in the values of T_g_^HIPS^ may suggests that the oil CNSL was not completely incorporated in PP phase and thus the following possibilities can be raised: 1. CNSL could be distributed throughout the two phases; 2. It could be incorporated only in HIPS phase; 3. It could be placed preferentially in the interface of the two phases. In Figure 15, the 3:2 blends, which have the lowest amount of HIPS, one can see that T_g_^HIPS^ decay is more significant as compared with the other blends. This suggests that CNSL may have a higher tendency to be incorporated into the HIPS phase, so that as the amount of HIPS in the blend is reduced, the relative concentration of CNSL increases in relation to the HIPS mass, thus indicating its effect on T_g_^HIPS^ reduction even more.

Concerning T_m_^PP^, the values were determined from the temperature at which the maximum of the crystalline melting peak occurs, as seen in Figure 10, Figure 11, Figure 12, Figure 13 and Figure 14. Unlike T_g_^HIPS^, no relevant variation in T_m_^PP^ has been found considering all samples, thus suggesting that neither the blend with HIPS nor the presence of CNSL had influence on the formation of a PP crystalline phase.

**Figure 16:**
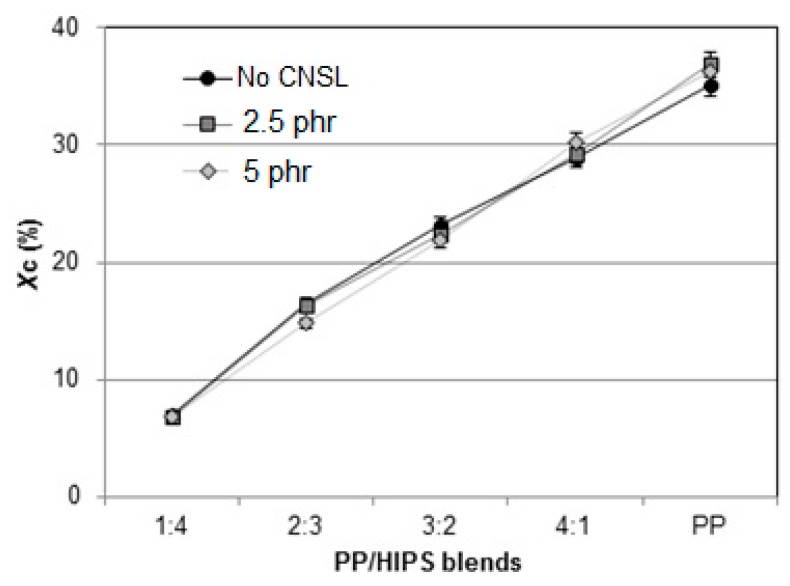
Degree of crystallinity $X_{c}$ of PP and the PP/HIPS blends with 0, 2.5 and 5 phr of CNSL.

The values of melting enthalpy $∆H_{f}$ for PP were obtained from the area under the melting peaks. No significant changes in enthalpy were observed in any sample on the addition of CNSL, as seen in Figure 10, Figure 11, Figure 12, Figure 13 and Figure 14, only a gradual reduction of these values as the amount of PP was also reduced. This can be better observed in Figure 16, which shows the calculated degree of crystallinity $X_{c}$ for the different blend compositions. An almost linear relationship between $X_{c}$ and the contribution of PP to the mixture was found, thus supporting the previous supposition that the presence of HIPS or CNSL does not affect the crystal formation of the PP phase.

### 3.3. Mechanical Tests

#### 3.3.1. Impact Strength Analysis

**Figure 17:**
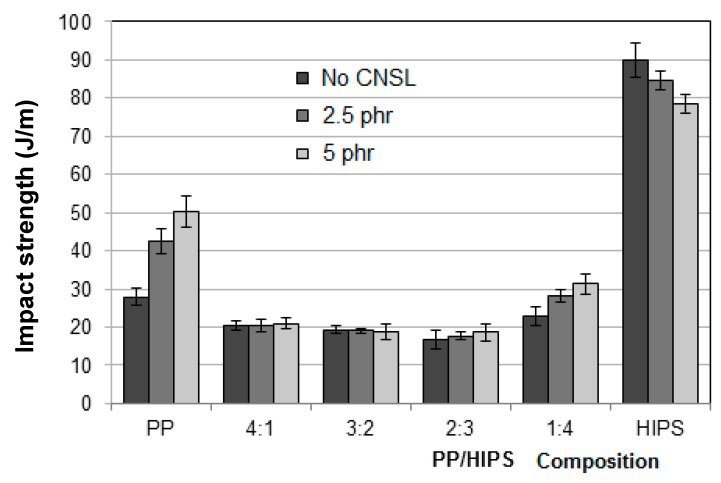
Impact strength results for PP, HIPS and PP/HIPS blends the PP/HIPS blends with 0, 2.5 and 5 phr of CNSL.

In general, impact strength is denoted as resilience, or the ability a material has to absorb mechanical energy. The values of impact strength for the neat polymers as well as the blends, in the presence or not of CNSL, are presented in Figure 17.

The neat polymers presented a visible effect on increasing CNSL addition but in opposite directions. PP experienced a gradual enlargement in impact resistance with the incorporation of a higher amount of CNSL, giving rise to an improvement of 80% in the property for the sample with 5 phr of CNSL, as compared with the sample with no oil. This can be due to a plasticizing effect of CNSL on the PP amorphous phase, thus increasing the efficiency of the sample in absorbing impact energy and improving its toughness property.

The adverse effect of CNSL on HIPS can be the result of a disturbing effect this substance may be imposing to HIPS morphology which would make the transfer of impact energy more difficult throughout the polymer matrix. The morphology formed during HIPS synthesis is such as to maximize the energy transfer. Any factor leading to modification in this morphology will cause, as consequence, a reduction in the impact strength.

Figure 17 also shows the results of impact resistance for the blends. The values for these samples are lower than for the neat PP, much lower than those for neat HIPS and very similar to each other, except for the 1:4 PP/HIPS blends. For the three first sets of blends, the deleterious effects of both PP phase and CNSL are well evidenced. Only the last set, with a larger amount of HIPS, could show a small recovery of the impact strength. As for the effect of PP, Parres et al. [9] showed that low levels of PP are sufficient to cause a drastic reduction of the impact resistance of HIPS, with losses as high as 40% by adding only 5% of PP.

The results can be corroborated by the effect of CNSL on the blends 1:4, previously seen in Figure 8. In the figure the stabilizing effect of CNSL was observed as the morphology of the blends became more homogeneous. Thus, the more uniform distribution of the domains of a phase throughout the other gave rise to a better absorption of the impact energy.

#### 3.3.2. Flexural Analysis

The flexibility of a material can be inferred from the elastic modulus determined in the tensile test, since it is directly related to rigidity. In spite of this, the flexural strength can be directly measured by the flexural application test itself, from which it is possible to obtain more appropriate results for the study of the flexibility of a material, that is, the capacity of the material to yield to a mechanical bending stress.

**Figure 18:**
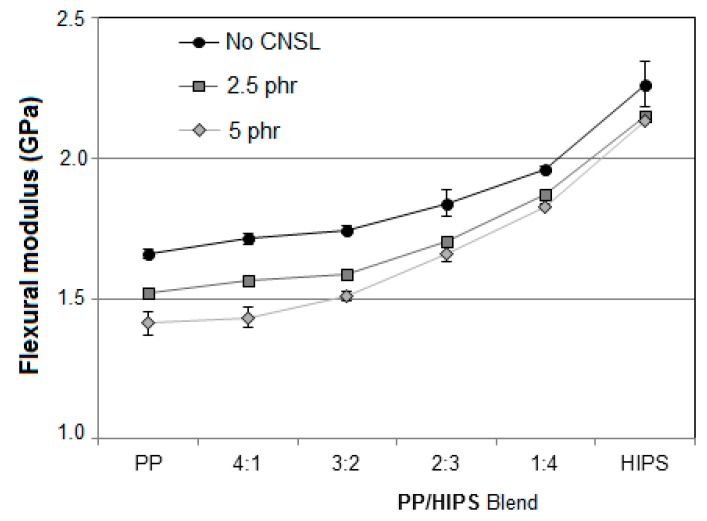
Flexural modulus for neat PP, HIPS and the PP/HIPS blends with 0, 2.5 and 5 phr of CNSL.

The results of flexural modulus are presented in Figure 18. Most error bars did not present a significant magnitude and some cannot be detected in the curves.

From these curves it is observed that increasing flexural modulus is directly related to the HIPS content in the samples, meaning that the higher the ratio between PP and HIPS, the lower the tension needed to promote a specific deformation under the bending stress.

On comparing the results related to the samples with and without CNSL, a reduction in the moduli was found as the oil content increased leading, in all cases, to an increase in material flexibility. This finding agrees with the idea that the CNSL molecules have been inserted in-between the polymer molecules thus creating secondary links, so that these molecules became separated from one another. This way, the cohesive forces among the macromolecules were reduced, thus promoting an increased mobility in the system and improving the flexibility of these materials. This intermolecular effect is characteristic of plasticizer additives [21,22], generally added to blends to increase flexibility, which was observed in the case of CNSL addition.

#### 3.3.3. Dynamic Melt Rheological Measurements

**Figure 19:**
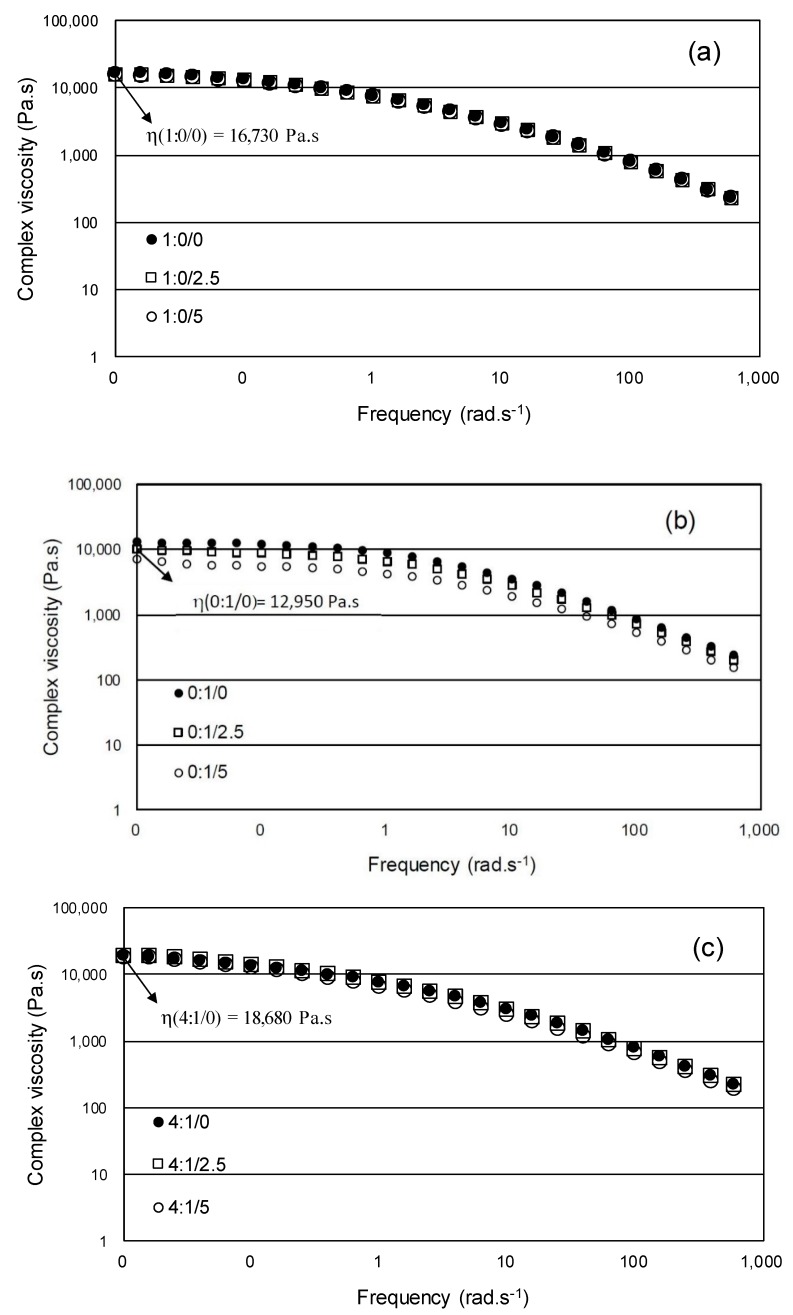

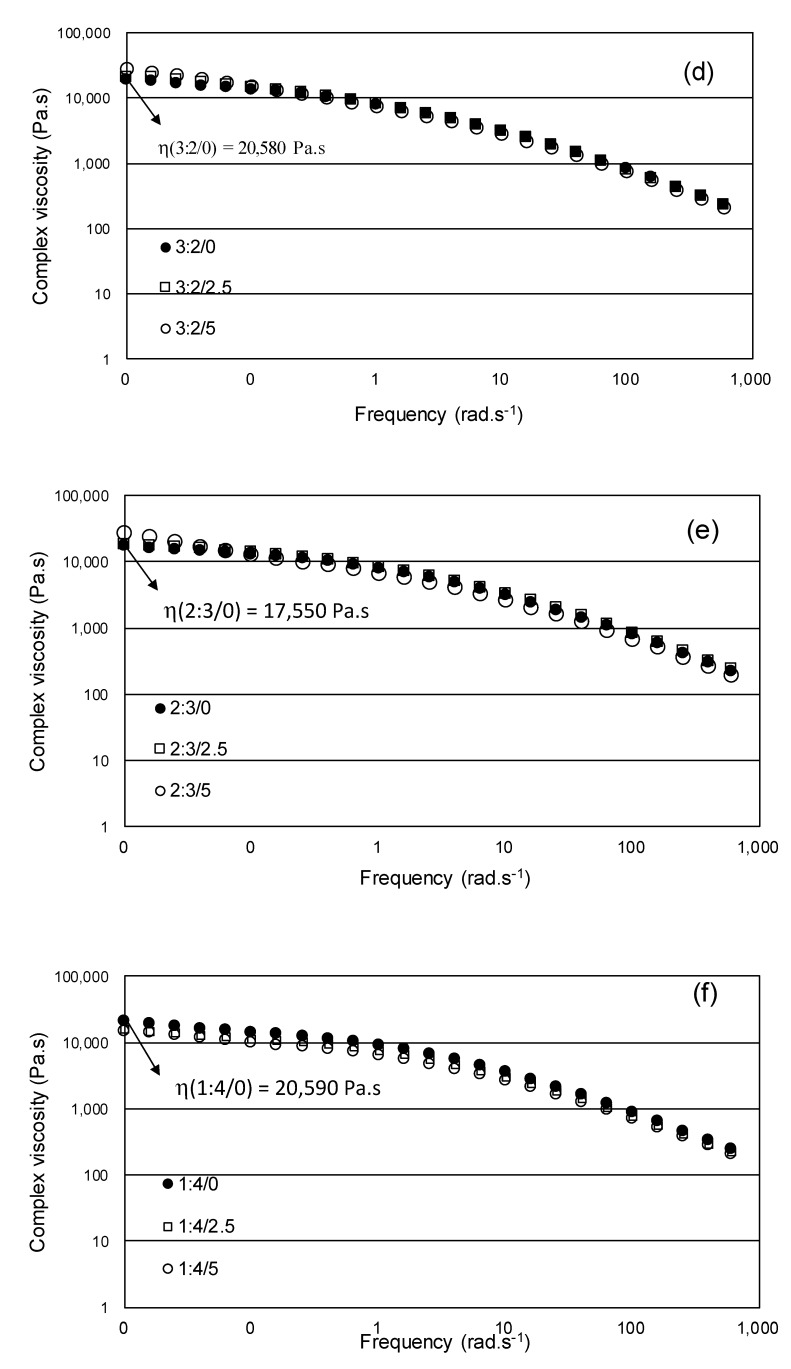
Complex viscosity *versus* frequency curves for: (**a**) PP; (**b**) HIPS and (**c**–**f**) 4:1, 3:2, 2:3 and 1:4 PP/HIPS blends, with 0, 2.5 and 5.0 phr CNSL content.

The variation of the complex shear viscosity as a function of the frequency of the neat PP, HIPS and PP/HIPS blends with different CNSL content is shown in Figure 19.

As seen in Figure 19, the neat PP and all the blends showed a shear-thinning behavior, with an increase in viscosity in the low-frequency region. Figure 19b shows that neat HIPS presents the lowest viscosity values in relation to neat PP and also a Newtonian plateau up to 0.1 rad·s^−1^ and then shows a shear-thinning behavior. It can also be observed that the addition of CNSL oil had a more pronounced effect on the flow behavior of HIPS in relation to PP. As HIPS is added to PP, an increase in viscosity values tends to occur due to the presence of HIPS domains which hinder the PP molecule flow. However, a different flow behavior occurred in 2:3/0 composition, that is, a decrease in viscosity values is observed in relation to other compositions without CNSL. SEM micrographs suggested that a co-continuous morphology occurred, which can explain the viscosity decrease in this blend. When CNSL oil is added to PP/HIPS blends, again it is observed a plasticizer effect, resulting in a more frequency-thinning behavior. As HIPS content increases in the blend composition, the oil tends to present a more pronounced plasticizer effect. It indicates that oil molecules interact more strongly with the HIPS phase, corroborating the SEM analysis. In 1:4/5 PP/HIPS/CNSL composition, the lowest viscosity values are observed, indicating once again that HIPS molecules achieve more mobility in the presence of the oil. This behavior corroborates the DSC results, which showed that the addition of CNSL oil reduces the Tg values of HIPS phase.

The variation of dynamic modulus, G’ and G”, was evaluated. Table 5 shows the values of the crossover point, at which G’ = G”, obtained from G’ and G” versus the frequency curves. The results were evaluated by the displacement of the G’ x G” crossover point, which allows the flow behavior of polymeric materials to be predicted [23].

Table 5 shows that the addition of HIPS to PP increases the elastic behavior of the blend and the addition of the CNSL oil tends to increase the viscous behavior, indicating its plasticizer effect in the PP/HIPS blends. This effect is more pronounced as HIPS content increases. A different behavior is observed in the 2:3/0 composition, which presents a decrease in the elastic behavior. As mentioned before, this blend showed a co-continuous morphology in SEM analysis. Once again, the rheological data show that the plasticizer effect of the oil is more pronounced in the HIPS phase.

Viscoelastic behavior from rheological measurements can be shown in a different representation called Cole-Cole plots. In this representation, the imaginary viscosity values (η” = G’/ω) is plotted against dynamic viscosity values (η’ = G”/ω). The plot should be a perfect arc in the absence of higher structures. In this case, the relaxation behavior of the melt will be described by a single relaxation time. Flattening of the arc, the presence of a tail or an increasing correlation indicate the occurrence of a broad relaxation time spectrum, while structural effects result in occurrence of a second arc [23,24].

**Figure 20:**
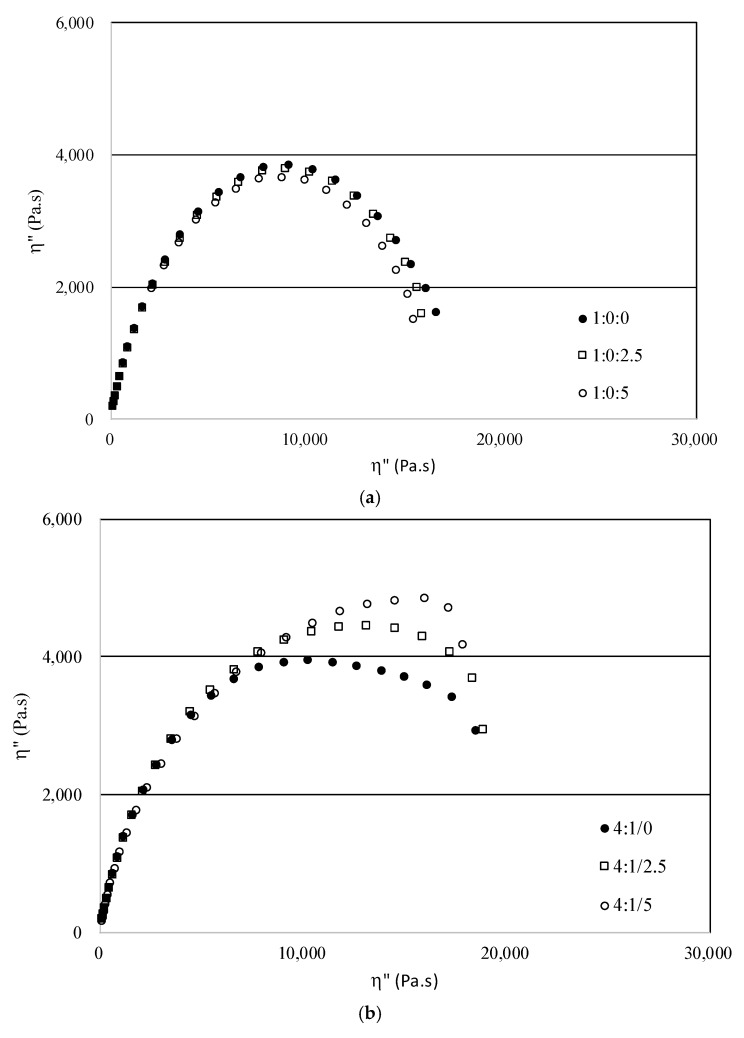

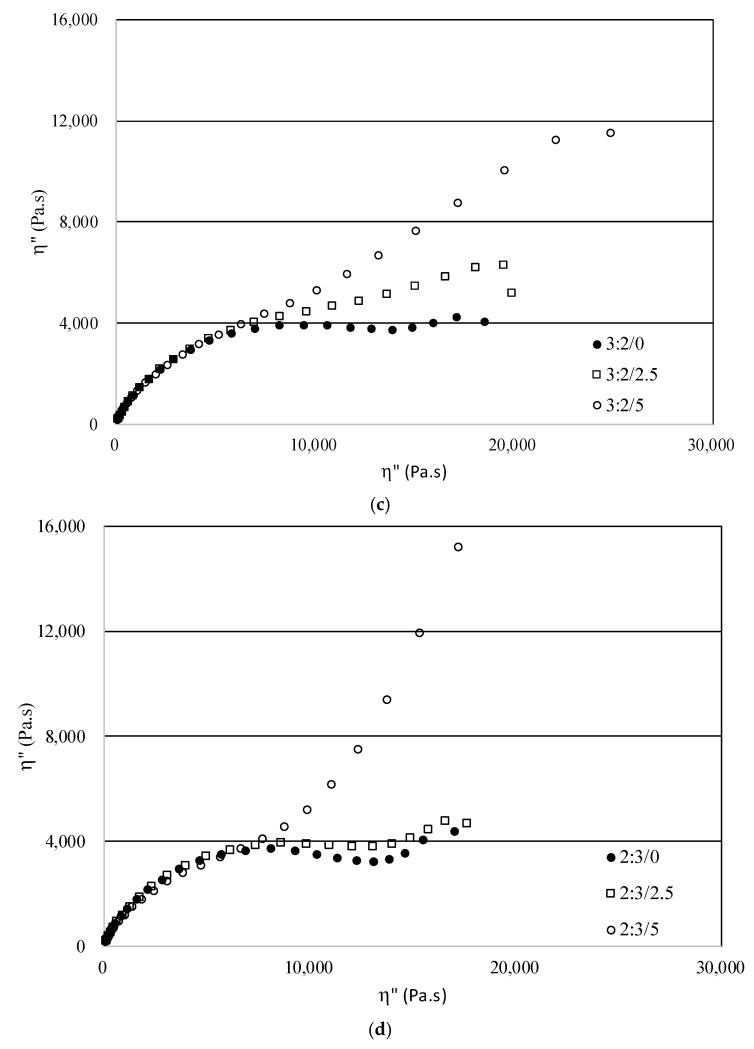

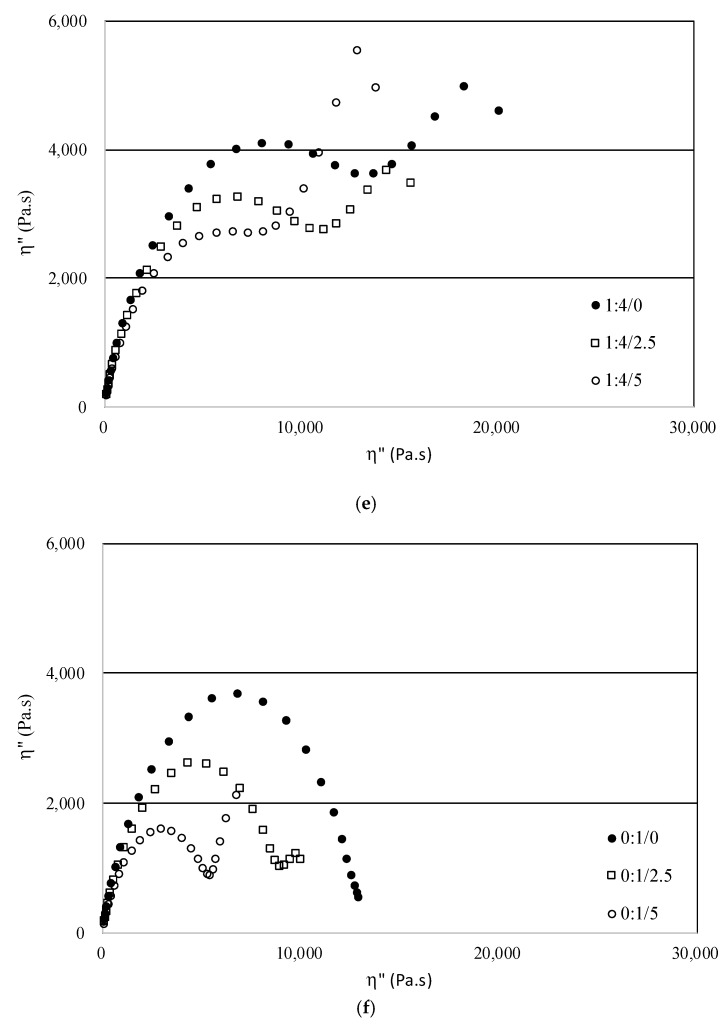
Cole-Cole plots of PP/HIPS blends (**a**) 1:0; (**b**) 4:1; (**c**) 3:2; (**d**) 2:3; (**e**) 1:4 and (**f**) 0:1, with different CNSL contents.

Figure 20 shows the Cole-Cole plots of the neat PP, HIPS and PP/HIPS blends with different CNSL content.

The Cole-Cole plot is like an arc for neat PP and PP/CNSL compositions, but deviates very strongly from an arc as HIPS and CNSL additive are added to PP, indicating a broad relaxation time due to the different interactions that occur between PP, HIPS and CNSL (when present) molecules. Figure 20f shows a perfect arc for neat HIPS and the appearance of arcs and tails for HIPS/CNSL compositions, indicating the occurrence of structural effects between HIPS and CNSL molecules. These results indicate the more pronounced effect of the CNSL oil on the HIPS phase.

## 4. Conclusions

The general evaluation of the morphological, mechanical and thermal aspects of the polypropylene (PP)/high impact polystyrene (HIPS) blends was carried out with the objective of investigating the effect of Cashew Nut Shell Liquid (CNSL) addition on these properties. Scanning Electron Microscopy (SEM) micrographies suggest that CNSL has a tendency to be located in the HIPS phase and also in the interface of the domains. This was corroborated by the results of Differential Scanning Calorimetry (DSC).

The addition of different contents of CNSL in PP/HIPS blends showed a general tendency to reduce the size of HIPS domains, thus suggesting a better interaction between the two polymers, providing tension transfer from one phase to the other and favoring stabilization of the morphology. Crystalline melting temperature of PP phase concerning all the blends did not change, thus corroborating the hypothesis that CNSL would be placed preferably in the HIPS phase or in the interphase between the two phases. Mechanical properties are in accordance with the proposed morphology, corroborated by rheological measurements. Cole-Cole plots also indicated that CNSL has a more pronounced effect on the HIPS phase.

## Figures and Tables

**Table 1:** Components of natural and technical CNSL [11].

Component	Natural CNSL (%)	Technical CNSL (%)
Anacardic acid	71–82	0–1.8
Cardanol	1.6–9.2	63–95
Cardol	13.8–20.3	3.8–18.9
2-Methyl-cardol	1.6–3.9	1.2–5.2
Polymeric material	-	0–21.63
Minor components	0–2.2	0–4

**Table 2:** Temperature profile used in the extrusion process.

Zone	1	2	3	4	5	6	7	8	9	Die
**Temperature (°C)**	90	140	150	150	160	165	170	175	180	180

**Table 3:** PP/HIPS compositions in the presence of different amounts of CNSL (0; 2.5; 5 phr).

PP/HIPS Ratio (%, *w*/*w*)	CNSL (phr)	Sample
1:0	0	1:0/0
	2.5	1:0/2.5
	5.0	1:0/5
4:1	0	1:0/0
	2.5	1:0/2.5
	5.0	1:0/5
3:2	0	4:1/0
	2.5	4:1/2.5
	5.0	4:1/5
2:3	0	3:2/0
	2.5	3:2/2.5
	5.0	3:2/5
1:4	0	2:3/0
	2.5	2:3/2.5
	5.0	2:3/5
0:1	0	1:4/0
	2.5	1:4/2.5
	5.0	0:1/5

**Table 4:** Injection parameters used in the processing.

Parameter	Zone				Die
	1	2	3	4	
Temperature (°C)	170	175	180	190	200
Injection pressure (bar)	1000				
Back pressure (bar)	300				

**Table 5:** Dynamic modulus and frequency values at the G’/G” crossover point for neat PP, HIPS and blends with 0, 2.5 and 5.0 phr CNSL contents.

PP/HIPS Ratio (%, *w*/*w*)	CNSL (phr)	Sample	Modulus at Crossover Point G’ = G” (Pa)	Crossover Point ω_c_ (rad/s)
1:0	0	1:0/0	20,670	10
	2.5	1:0/2.5	21,080	10
	5.0	1:0/5	20,780	10
4:1	0	4:1/0	21,010	10
	2.5	4:1/2.5	20,730	10
	5.0	4:1/5	17,700	10
3:2	0	3:2/0	22,120	10
	2.5	3:2/2.5	21,900	10
	5.0	3:2/5	19,910	10
2:3	0	2:3/0	21,600	10
	2.5	2:3/2.5	22,940	10
	5.0	2:3/5	18,260	10
1:4	0	1:4/0	25,130	10
	2.5	1:4/2.5	21,620	10
	5.0	1:4/5	18,030	10
0:1	0	0:1/0	25,280	10
	2.5	0:1/2.5	20,250	10
	5.0	0:1/5	17,270	16
